# Phase 3 Trial of BI 695502 Plus Chemotherapy Versus Bevacizumab Reference Product Plus Chemotherapy in Patients With Advanced Nonsquamous NSCLC

**DOI:** 10.1016/j.jtocrr.2021.100248

**Published:** 2021-10-28

**Authors:** Edward S. Kim, Sigrid Balser, Klaus B. Rohr, Ragna Lohmann, Bernd Liedert, Dorothee Schliephake

**Affiliations:** a City of Hope National Medical Center, Los Angeles, California; b Boehringer Ingelheim Pharma GmbH & Co. KG, Ingelheim, Germany; c Boehringer Ingelheim International GmbH, Ingelheim, Germany

**Keywords:** BI 695502, Bevacizumab, Biosimilar, Non–small-cell lung cancer

## Abstract

**Introduction:**

Biological therapies such as bevacizumab have improved survival in patients with NSCLC. This study was conducted to confirm the equivalent efficacy of the biosimilar candidate BI 695502 to the bevacizumab reference product (RP).

**Methods:**

In this phase 3, multicenter, randomized, double-blind trial of adult patients with recurrent or metastatic NSCLC received up to 18 weeks of induction treatment with BI 695502 or bevacizumab RP 15 mg/kg plus paclitaxel and carboplatin. Subsequent maintenance therapy comprised BI 695502 or bevacizumab RP monotherapy until disease progression or unacceptable toxicity. The primary end point was the best overall response rate (ORR) per Response Evaluation Criteria in Solid Tumors version 1.1 assessed by central imaging review, until 18 weeks after the start of treatment.

**Results:**

In total, 671 patients were randomized at one-to-one ratio to BI 695502 or bevacizumab RP, of whom 335 and 328, respectively, received treatment. Of these, 228 (68.1%) and 256 (78.0%), respectively, proceeded to maintenance monotherapy. A manufacturing issue led to a small number of patients treated with BI 695502 switching to bevacizumab RP late in the study. The primary end point, best ORR, was 54.0% in the BI 695502 group and 63.1% in the bevacizumab RP group. The 90% confidence interval for the between-group ratio of best ORR (0.770 to 0.951) was within the prespecified range for equivalence (0.736–1.359). Adverse events were class-related and similar between the two treatment arms.

**Conclusions:**

This study revealed equivalent ORR after 18 weeks of treatment with BI 695502 or bevacizumab RP, with similar adverse event profiles.

## Introduction

Lung cancer has the highest global incidence and mortality of all cancer types.^1^ For 2018, more than 2 million new cases were predicted globally, and over 1.7 million deaths.^1^ A wide variety of treatments are available for lung cancer, including biological therapies (or biologics). These relatively new agents have improved survival outcomes in patients with advanced NSCLC,^2^ which is the most common type of lung cancer.^3^ Bevacizumab is a recombinant humanized monoclonal antibody directed against vascular endothelial growth factor (VEGF),^4^^,^^5^ which plays a key role in tumor growth and metastasis through the promotion of angiogenesis and neovascularization.^6^ A number of key phase 2 to 4 clinical trials,^10^, ^11^, ^12^, ^7^, ^8^, ^9^ and meta-analyses,^13^, ^14^, ^15^ have confirmed that adding bevacizumab to chemotherapy improves survival in NSCLC and other solid tumors. Bevacizumab is approved in the United States (Avastin; Genentech, CA) and European Union (EU) (Avastin; Roche, Welwyn Garden City, United Kingdom) as a treatment for nonsquamous NSCLC and a range of other cancer types.^16^, ^17^, ^18^

Increasing numbers of biosimilar drugs are becoming available as alternatives to the original patented biologics. Biosimilars are developed to be structurally and functionally similar to the reference product (RP), so they may be used the same way in clinical practice.^5^^,^^19^ To gain regulatory approval, biosimilars must first demonstrate bioequivalence with respect to pharmacokinetics (PK) and pharmacodynamics. Equivalent efficacy, safety, and immunogenicity with the RP must then be demonstrate in at least one clinical study.^5^^,^^19^ Biosimilars may be approved for multiple RP indications even if clinical data are not available for each one on the basis of the principle of indication-extrapolation.^5^^,^^20^^,^^21^

As of February 2021, a total of 29 biosimilars had been approved in the United States. These included six adalimumab (Humira; AbbVie, Lake Bluff, IL), five trastuzumab (Herceptin; Genentech, South San Francisco, CA), four pegfilgrastim (Neulasta; Amgen, Thousand Oaks, CA), and four infliximab (Remicade; Janssen, Horsham, PA) biosimilars.^22^ Patents for Avastin expired in the United States in 2019 and will expire in the EU in 2022, and considerable effort has been made to develop biosimilars for this drug.^5^^,^^21^ Two biosimilars to bevacizumab RP have been approved to date (Zirabev [Pfizer, New York, NY] and Mvasi [Amgen]) for multiple indications, including unresectable, locally advanced NSCLC and recurrent or metastatic nonsquamous NSCLC.^23^^,^^24^

BI 695502 was developed as a bevacizumab biosimilar candidate but is no longer under development by Boehringer Ingelheim. A phase 1 trial in healthy male subjects demonstrated three-way PK similarity of BI 695502 to the U.S.-licensed and EU-approved bevacizumab RP.^25^ The study reported here was conducted to establish equivalence in terms of efficacy for BI 695502 and U.S.-licensed bevacizumab RP in patients with advanced nonsquamous NSCLC when given as induction therapy in combination with chemotherapy.

## Materials and Methods

### Study Design and Treatment

This was a phase 3, multicenter, randomized, double-blind, parallel-group, active comparator trial (clinicaltrials.gov identifier: NCT02272413). Patients were randomized in a one-to-one ratio to receive either BI 695502 or bevacizumab RP (U.S.-licensed Avastin) (Supplementary Fig. 1). The study consisted of induction therapy lasting for up to 18 weeks followed by maintenance therapy until disease progression per Response Evaluation Criteria in Solid Tumors version 1.1 (RECIST v1.1), death, withdrawal of consent, unacceptable toxicity, or the end of the study. Randomization was stratified by sex, smoking status, disease stage, and ethnicity (East Asian versus non-East Asian origin). Randomization was performed by means of an interactive telephone and web response system (IXRS; Almac Clinical Technologies, Souderton, PA), with each patient being assigned a unique number. Study blinding was maintained by ensuring that no individuals directly involved in the conduct or analysis of the trial had access to treatment allocation details before the final database lock. As the primary analysis was based only on the induction period, it was scheduled before completion of the study; the required data were unblinded only for individuals performing the primary data analyses.

Induction therapy comprised BI 695502 or bevacizumab RP 15 mg/kg followed by standard combination chemotherapy of paclitaxel 200 mg/m^2^ body surface area (administered according to regular institutional practice) followed by carboplatin target area under the concentration curve 6 mg/mL per min (30–60 min infusion), with adequate pre and concomitant medication, every 3 weeks (each cycle) for up to six cycles. Maintenance therapy (monotherapy with BI 695502 or bevacizumab RP as per the original randomization) was given to patients who did not have disease progression per RECIST v1.1 (i.e., had complete response [CR], partial response, or stable disease) after up to six induction cycles.

After a manufacturing issue with a single batch of BI 695502 in December 2017, when recruitment was complete and 87% of patients had already completed maintenance, investigators were asked to switch the remaining 13% of patients (all of whom were in the maintenance phase) from BI 695502 to bevacizumab RP (Supplementary Fig. 1). Comparisons of BI 695502 with bevacizumab RP were primarily on the basis of the data obtained before the treatment switch (i.e., during the preswitch period).

### Patients and Ethics

Patients aged at least 18 years (≥20 y in Japan) with histologically or cytologically confirmed nonsquamous NSCLC were eligible to participate in the study, provided their disease was recurrent or metastatic (stage IV) and were suitable for treatment with paclitaxel, carboplatin, and bevacizumab. In addition, patients had to have at least one measurable lesion (in accordance with RECIST v1.1) and an Eastern Cooperative Oncology Group performance status (ECOG PS) of 0 or 1. Patients were excluded if they had received previous therapy with monoclonal antibodies or small-molecule VEGF inhibitors, or previous systemic therapy for metastatic disease. Comprehensive inclusion and exclusion criteria are provided as Supplementary Material.

The protocol and other documents relating to the study were approved by the applicable institutional review boards and independent ethics committees. The principles of the Declaration of Helsinki and the International Council for Harmonization of Technical Requirements for Pharmaceuticals for Human Use tripartite guidelines for Good Clinical Practice were adhered to throughout. All patients provided written informed consent before participating.

### Assessments

Clinic visits were scheduled every 3 weeks throughout the induction and maintenance treatment periods, for the administration of study medication and performance of study assessments. Patients attended further visits at the end of treatment and, for safety follow-up, 18 weeks after the last dose of study medication.

The primary end point was the best overall response rate (ORR) (either CR or partial response) 0 to 18 weeks after the start of treatment, as assessed per RECIST v1.1 by central imaging review. Tumor assessment was not confirmed by a subsequent assessment. BI 695502 was to be deemed equivalent to bevacizumab RP if the 2-sided 90% confidence interval (CI) for the between-group ratio of best ORR (BI 695502/bevacizumab RP) was within the range 0.736 to 1.359. This margin was based on a meta-analysis of three historical studies of the bevacizumab RP.^8^^,^^9^^,^^12^ An additional determination, on the basis of whether the 95% CI was within the range 0.727 to 1.376, was made to fulfill regulatory requirements in Japan and Europe.

Secondary efficacy end points were overall survival (OS), and progression-free survival (PFS) and duration of response (DoR) assessed by investigators according to RECIST v1.1 during the preswitch period. Safety end points included percentages of patients with adverse events (AEs: all-causality, treatment-related, grade 3/4, immunogenicity-related). Incidence rates for AEs of special interest (hepatic injury, gastrointestinal [GI] perforations, anaphylactic reactions, and pulmonary hemorrhage) were also calculated. A comparison of the safety of BI 695502 and bevacizumab RP was conducted on the basis of the proportions of patients with preselected AEs in nine categories.

PK analyses were performed using plasma from blood samples obtained during treatment and at follow-up. To assess the immunogenicity of each treatment, the proportions of patients with antidrug antibodies (ADAs) and neutralizing ADAs (nAbs) were calculated.

### Statistical Analyses

The sample size calculation was based on the assumption that the ORR would be 43.3% in both treatment arms and that the between-group ratio of best ORR would be 1.000. To provide a power of 92% to test the primary hypothesis (i.e., that the ratio for best ORR by week 18 [BI 695502 versus bevacizumab RP] would be within the interval of 0.736–1.359), a total of 660 patients (330 per treatment arm) would be required. After approximately 200 patients (100 patients per arm) had tumor response assessments performed until week 18, an independent blinded statistician performed a blinded evaluation to confirm the calculated sample size; the blinded sample size review revealed that no changes were needed.

Efficacy analyses were performed using the full analysis set (FAS), which comprised all randomized patients who had a baseline tumor assessment and received at least one dose of study medication. Individuals in the FAS with no important protocol violations comprised the per-protocol set. The treated set (all randomized patients who received at least one dose of study medication) was used for safety and immunogenicity assessments. All patients who received at least 1 dose of study medication and for whom at least one measurement of study drug concentration was available were included in the population for PK analysis.

For the primary efficacy analysis, data were collected until the central imaging review of all week 18 tumor assessments had been completed, or earlier if no more patients were expected to complete the week 18 visit. If a patient started an alternative anticancer therapy (not specified by the protocol) during the induction period, the best ORR was evaluated only until the alternative therapy was initiated. The statistical model was based on a logarithmic binomial regression model with subsequent transformation to the ratio of proportions. A range of prespecified sensitivity analyses was conducted in the per-protocol set and in the FAS, some of which were on the basis of different equivalence margins (details are provided in Supplementary Table 1).

PFS, OS, and DoR were analyzed using a Cox-proportional hazards model using the same adjustment factors as for best ORR. PK end points were analyzed using descriptive statistics. AEs were coded using the Medical Dictionary for Regulatory Activities (version 21.1) and graded according to National Cancer Institute Common Terminology Criteria for Adverse Events (version 4.0).

## Results

### Patients

**Figure 1:**
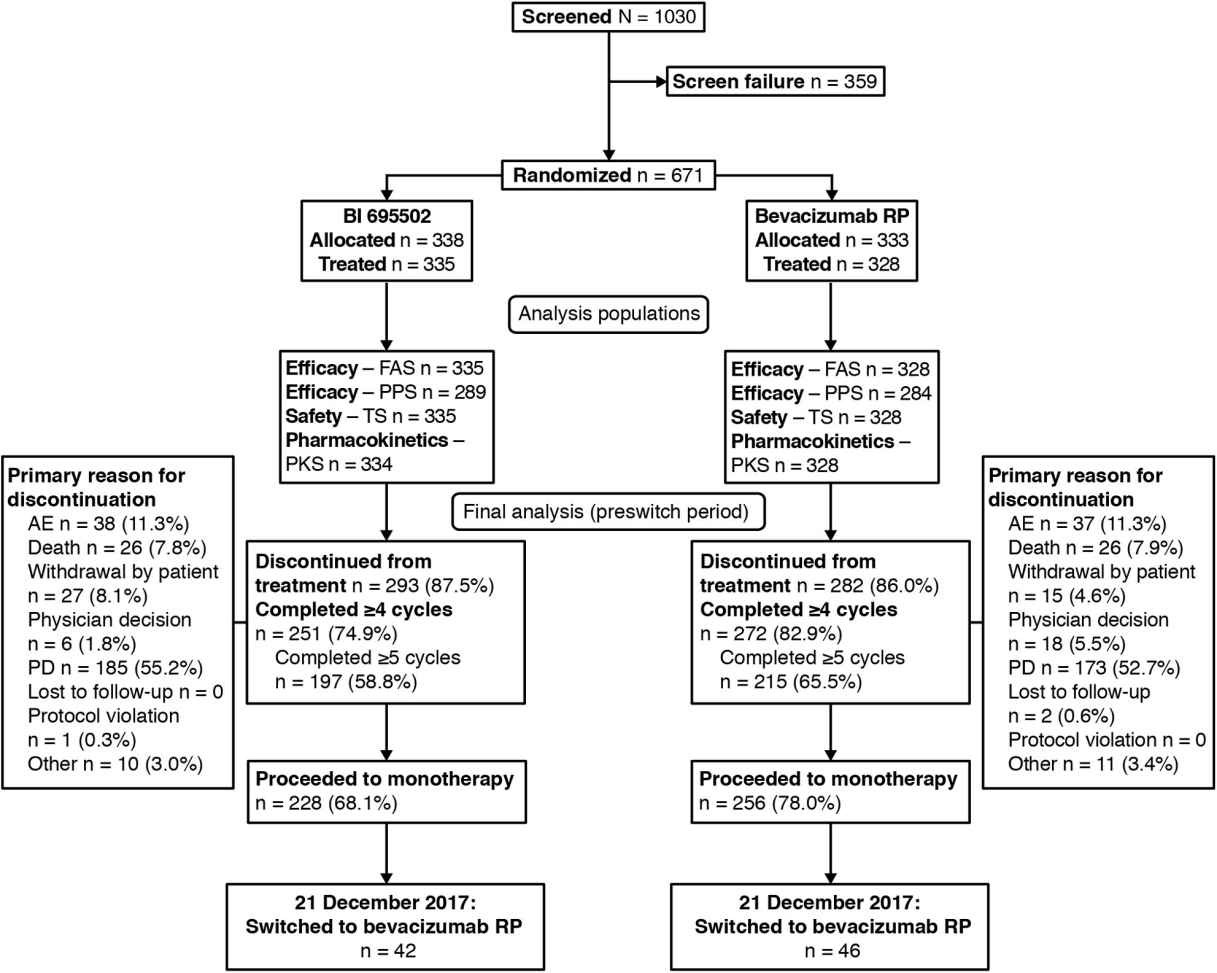
Patient disposition. AE, adverse event; FAS, full analysis set; PD, progressive disease; PKS, pharmacokinetic analysis set; PPS, per-protocol analysis set; RP, reference product; TS, treated set.

A total of 1030 patients were screened at 190 trial centers, and 671 patients from 161 centers in 28 countries were randomized (Fig. 1). The first patient was screened on July 21, 2015, and the last visit of the last patient was on November 16, 2018. Demographic and baseline characteristics were similar across treatment arms and were representative of the intended target patient population (Table 1). The study population included more males than females (62.9% versus 37.1%), and the predominant race was White (76.3%). Most patients (91.7%) had stage IV NSCLC at screening. A higher proportion of patients in the BI 695502 group (6.6%) had nontarget brain lesions compared with the bevacizumab RP group (3.7%).

**Table 1:** Patient Demographics and Baseline Characteristics (FAS)

	BI 695502 (n = 335)	Bevacizumab RP (n = 328)
Age, mean (SD), years	61.2 (9.89)	61.3 (9.22)
Male sex, n (%)	214 (63.9)	203 (61.9)
Race, n (%)		
White	258 (77.0)	248 (75.6)
Asian	64 (19.1)	71 (21.6)
Black or African American	1 (0.3)	1 (0.3)
Other	12 (3.6)	8 (2.4)
Ethnicity, n (%)		
East Asian	64 (19.1)	68 (20.7)
Non-East Asian	271 (80.9)	260 (79.3)
Current/ex-smoker, n (%)	237 (70.7)	230 (70.1)
Time since diagnosis of lung cancer, median (Q1, Q3), months	1.12 (0.62, 2.43)	0.94 (0.49, 1.69)
Cancer stage at screening, n (%)		
Recurrent	28 (8.4)	27 (8.2)
Metastatic stage IV	307 (91.6)	301 (91.8)
ECOG performance status, n (%)		
0	124 (37.0)	130 (39.6)
1	211 (63.0)	198 (60.4)
Brain lesions (nontarget lesion), n (%)	22 (6.6)	12 (3.7)

ECOG, Eastern Cooperative Oncology Group; FAS, full analysis set; Q, quartile; RP, reference product; SD, standard deviation.

### Efficacy

In the primary analysis, the best unconfirmed ORR was 54.0% in the BI 695502 group and 63.1% in the bevacizumab RP group (Table 2). Equivalence was exhibited because the 90% CI for the best ORR ratio (0.770–0.951) was within the prespecified range of 0.736 to 1.359. The 95% CI for the ratio of best ORR (0.754–0.970) also fell within the required range, satisfying the Japanese and European criteria for equivalence. As detailed in Supplementary Table 1, all prespecified sensitivity analyses indicated equivalence between BI 695502 and bevacizumab RP, as CIs were within the prespecified equivalence margins. An additional sensitivity analysis of the difference in the best ORR until 18 weeks after the start of treatment for BI 695502 minus bevacizumab RP was conducted using the FAS. No equivalence margins were predefined for this analysis. The observed difference was −9.08% (90% CI: −15.439% to −2.638%; 95% CI: −16.637% to −1.415%) and the difference after adjustment for treatment, sex, smoking status, disease stage, and (non-)East Asian origin was −8.89% (90% CI: −16.338% to −1.872%; 95% CI: −17.729% to −0.518%).

**Table 2:** Primary Efficacy Analysis (FAS)

	BI 695502 (n = 335)	Bevacizumab RP (n = 328)
Best ORR (CR + PR), observed, n (%) Best ORR (CR + PR), adjusted,^a^ % (95% CI)	181 (54.0) 55.9 (48.2–64.8)	207 (63.1) 65.3 (56.4–75.6)
Ratio of best ORR 90% CI	0.855 0.770–0.951	
95% CI^b^	0.754–0.970	
Best response until Week 18, n (%)		
CR	3 (0.9)	2 (0.6)
PR	178 (53.1)	205 (62.5)
SD	96 (28.7)	91 (27.7)
PD	27 (8.1)	10 (3.0)
Not evaluable	0	1 (0.3)
Missing	31 (9.3)	19 (5.8)

CI, confidence interval; CR, complete response; FAS, full analysis set; ORR, overall response rate; PD, progressive disease; PR, partial response; RP, reference product; SD, stable disease.

a Adjusted for treatment, sex, smoking status, NSCLC stage and (non-)East Asian origin.

b Additional analysis for EU and primary analysis for Japan.

**Figure 2:**
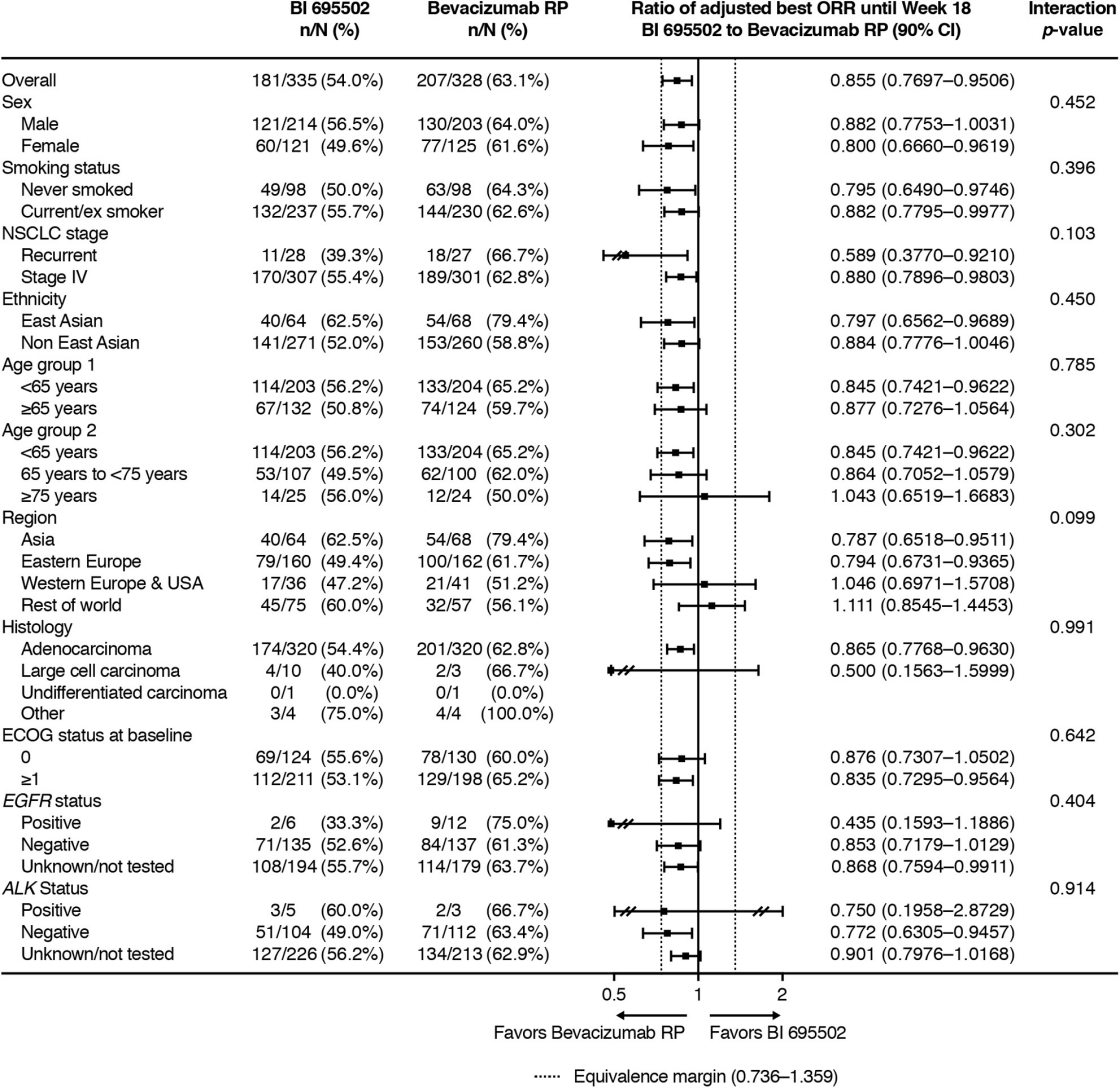
Best overall response ratios: subgroup analyses. CI, confidence interval; ECOG, Eastern Cooperative Oncology Group; ORR, overall response rate; RP, reference product.

Subgroup analyses of the primary end point revealed that the outcome was largely consistent across different patient subgroups, including sex, smoking status, ethnicity, age, geographic region, and baseline ECOG PS (Fig. 2). Investigator-assessed best ORR from 0 to 18 weeks was also similar between BI 695502 and bevacizumab RP, at 47.5% (159 of 335) in the BI 695502 group and 52.1% (171 of 328) in the bevacizumab RP group (ratio = 0.908, 90% CI: 0.799–1.032).

Best responses during weeks 0 to 18 are detailed in Table 2. CR was achieved by less than 1% of patients in each treatment group. Similar proportions of patients in each treatment group had stable disease, whereas a higher proportion of patients in the BI 695502 group than in the bevacizumab RP group had progressive disease (PD).

The investigator-assessed median DoR (on the basis of Kaplan–Meier estimates) was 7.7 months with BI 695502 and 8.9 months with bevacizumab RP (HR = 1.14, 95% CI: 0.88–1.48). By the end of the preswitch period, similar proportions of patients in both treatment groups had PD (BI 695502, 114 [65.1%]; bevacizumab RP, 124 [66.3%]).

**Figure 3:**
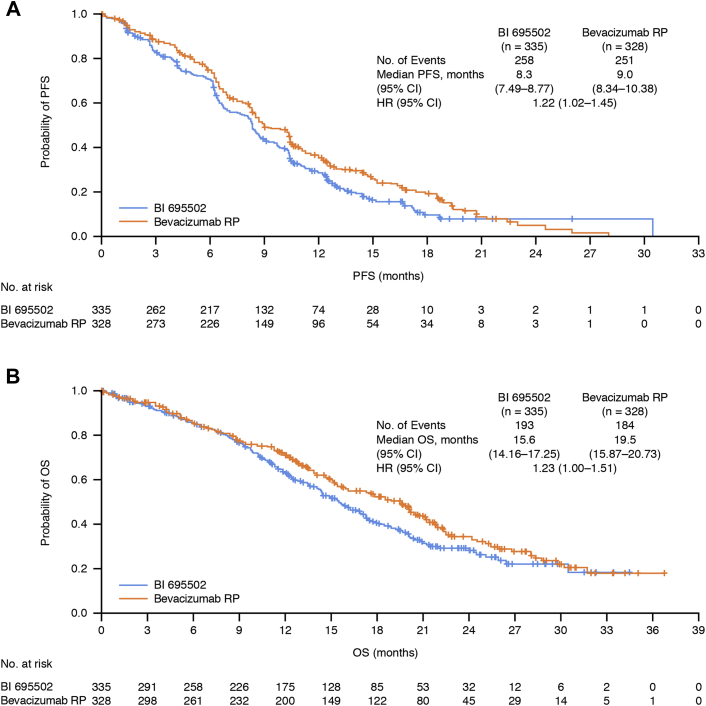
Kaplan–Meier curves for the preswitch period: *(A)* PFS and *(B)* OS. CI, confidence interval; HR, hazard ratio; OS, overall survival; PFS, progression-free survival; RP, reference product.

Kaplan–Meier curves for PFS and OS during the preswitch period revealed similar patterns in the two study arms, although there was evidence of a trend toward slightly improved outcomes with bevacizumab RP (Fig. 3). The median PFS was 8.3 months in patients receiving BI 695502 and 9.0 months in those treated with bevacizumab RP (HR = 1.22, 95% CI: 1.02–1.45). The median OS was 15.6 months in the BI 695502 treatment group and 19.5 months in the bevacizumab RP group (HR = 1.23, 95% CI: 1.00–1.51).

### PK

After a total of 90 minutes from the first infusion of the study drug, the median plasma level of BI 695502 was 290 μg/mL (interquartile range [IQR]: 244–363 μg/mL), compared with 305 μg/mL (IQR: 258–374 μg/mL) with bevacizumab RP. Preinfusion plasma concentrations of both study drugs increased gradually over time (Supplementary Fig. 2). Plasma concentrations were slightly higher in the bevacizumab RP group than in the BI 695502 group. However, the differences were small and not considered to be clinically relevant.

### Immunogenicity

Among patients with negative or unknown ADA status at baseline, four patients (1.2%) in the BI 695502 group, and eight patients (2.4%) in the bevacizumab RP group had at least one positive ADA result postbaseline. All ADA-positive samples were negative for nAbs. The switch from BI 695502 to bevacizumab RP had no impact on the percentages of patients with ADAs or nAbs (data not presented).

### Safety

The proportions of patients with at least one AE in the BI 695502 and bevacizumab RP groups were similar during the preswitch period, as were the proportions of patients with drug-related AEs (Table 3). The most common AEs (those reported in ≥10% of patients) were similar across both treatment groups and occurred at similar frequencies in each group (although the frequency was slightly higher in the BI 695502 group). The most common AE in both groups was alopecia (BI 695502, 46.3%; bevacizumab RP, 45.4%).

**Table 3:** Safety Summary (Treated Set)

AEs, n (%)	BI 695502 (n = 335)	Bevacizumab RP (n = 328)
Any AE	312 (93.1)	313 (95.4)
Related to study drug	181 (54.0)	172 (52.4)
AEs occurring in ≥10% of patients in either treatment group		
Alopecia	155 (46.3)	149 (45.4)
Anemia	113 (33.7)	89 (27.1)
Nausea	74 (22.1)	76 (23.2)
Neutropenia	64 (19.1)	58 (17.7)
Peripheral neuropathy	62 (18.5)	59 (18.0)
Diarrhea	60 (17.9)	48 (14.6)
Vomiting	59 (17.6)	38 (11.6)
Fatigue	54 (16.1)	56 (17.1)
Decreased appetite	54 (16.1)	55 (16.8)
Peripheral sensory neuropathy	56 (16.7)	53 (16.2)
Hypertension	49 (14.6)	51 (15.5)
Constipation	52 (15.5)	44 (13.4)
Proteinuria	51 (15.2)	46 (14.0)
Thrombocytopenia	45 (13.4)	50 (15.2)
Neutrophil count decreased	41 (12.2)	42 (12.8)
Platelet count decreased	43 (12.8)	35 (10.7)
Arthralgia	40 (11.9)	35 (10.7)
Epistaxis	38 (11.3)	33 (10.1)
Cough	38 (11.3)	32 (9.8)
Myalgia	38 (11.3)	29 (8.8)
Dyspnea	27 (8.1)	34 (10.4)
Any AE grade 3/4	164 (49.0)	154 (47.0)
Related to study drug	73 (21.8)	58 (17.7)
Any SAE	108 (32.2)	89 (27.1)
Related to study drug	42 (12.5)	27 (8.2)
AE leading to death	22 (6.6)	17 (5.2)
Related to study drug	4 (1.2)	2 (0.6)
Any AE potentially related to immunogenicity	1 (0.3)	0
Any investigator-reported AESI^a^	12 (3.6)	3 (0.9)
Any AE leading to study drug discontinuation	40 (11.9)	38 (11.6)

AE, adverse event; AESI, AE of special interest; RP, reference product; SAE, serious adverse event.

a AESIs included: hepatic injury, anaphylactic reactions, gastrointestinal perforations, pulmonary hemorrhage.

All-causality and drug-related grade 3/4 AEs occurred in a higher proportion of patients in the BI 695502 group compared with the bevacizumab RP group. However, these differences were small. A similar pattern was observed in the frequencies of serious AEs (SAEs). AEs leading to death were reported in a small number of patients in each treatment group. These AEs were drug-related in four patients in the BI 695502 group and two patients in the bevacizumab RP group.

Signs and symptoms of immunogenicity were rare and there was no discernible difference between the treatment groups in this regard. Only one study participant, who was receiving BI 695502, had AEs identified as potentially related to immunogenicity. These AEs were dyspepsia and vomiting; both events were nonserious and grade 1. AEs of special interest (hepatic injury, anaphylactic reactions, GI perforations, pulmonary hemorrhage) were reported in a higher percentage of patients in the BI 695502 group (3.6%) compared with the bevacizumab RP group (0.9%). GI perforations and pulmonary hemorrhage were seen in 2.1% and 1.2% of patients treated with BI 695502 and 0.6% and 0.9% of patients treated with bevacizumab RP. The proportion of patients with AEs selected for the comparability assessment was also higher in the BI 695502 group (52.5%), as compared with the bevacizumab RP group (45.1%). However, the 95% CI of the risk ratio (BI 695502/bevacizumab RP; the calculated value of 1.16) was 0.99 to 1.37 and the inclusion of one within this range indicates that the two treatments were comparable. The 95% CIs for all nine categories of AEs selected for comparison also encompassed 1.

During the postswitch period, AEs occurred in similar proportions of patients switching from BI 695502 to bevacizumab RP as in patients receiving bevacizumab RP throughout the study (59.5% versus 56.5%).

## Discussion

Biosimilars offer many benefits to both patients and payors. In addition to increasing the number of available treatment options and offering comparable treatment at a lower cost, these agents may facilitate increased access to effective therapies. Studies like the one reported here are, therefore, integral to the increased use of biosimilars across a range of indications. Our study revealed equivalence between BI 695502 and bevacizumab RP, with the 90% CI for the primary efficacy end point (best ORR until 18 wks) lying within the prespecified range. This result was robust, as exhibited by sensitivity analyses, with the criteria for equivalence being fulfilled consistently. Overall safety results were also similar between the two treatment arms, and immunogenicity was low with both BI 695502 and bevacizumab RP. Numerically, BI 695502 seemed to perform slightly less favorably than bevacizumab RP in terms of the proportion of patients who experienced PD (0–18 wks), PFS (preswitch period), and SAEs.

PK analysis indicated that plasma concentrations were slightly higher with bevacizumab RP than BI 6595502 (Supplementary Fig. 2). However, IQRs were largely overlapping, indicating a lack of statistical significance, and the small magnitude of the differences suggests no clinical significance. In a previous study comparing BI 695502 with two RPs (U.S.- and EU-approved bevacizumab RP) in healthy volunteers,^25^ results for the primary end point of PK (area under the concentration-time curve from time zero extrapolated to infinity) and all secondary PK parameters met the criteria for three-way bioequivalence^25^ even though the exposure was lower in the BI 695502 group than in both bevacizumab RP groups in this phase 1 study. Although PK equivalence testing was not performed in this study, the results seem consistent with those of the previous study.

It is possible that small differences in plasma concentrations could affect efficacy and/or toxicity. However, a meta-analysis of bevacizumab RP clinical trials found no meaningful difference in PFS at doses of between 7.5 and 15 mg/kg.^26^ Toxicity data from the same meta-analysis suggest that increased exposure to the drug could increase the risk of AEs. This pattern was not observed in the present study, as plasma exposure was higher with the bevacizumab RP but grade 3/4 AEs and SAEs were slightly more common in the BI 69502 group. Therefore, the small differences we observed in drug plasma concentrations do not seem to explain the variations in efficacy or toxicity. Baseline demographic and disease characteristics were generally similar between the two treatment groups; the only notable difference being that slightly more patients in the BI 695502 group had ECOG PS of 1 (63.0% versus 60.4%), and slightly more patients had brain lesions (6.6% versus 3.7%) than in the bevacizumab RP group. These differences suggest that patients in the BI 695502 may have been in slightly worse condition than the bevacizumab RP group before starting therapy, and, therefore, less likely to respond to treatment and more likely to experience AEs.

Similar efficacy and toxicity have been observed in clinical studies of other bevacizumab biosimilars compared with bevacizumab RP. Equivalence for the bevacizumab biosimilar PF-06439535 (Zirabev) and EU-approved bevacizumab RP as first-line treatment of advanced nonsquamous NSCLC, in combination with paclitaxel and carboplatin, was demonstrated in a randomized double-blind study of 719 patients.^27^ The ORR by week 19 was 45.3% in the PF-06439535 group and 44.6% in the bevacizumab-EU group. This is slightly lower than the ORRs of 54.0% in the BI 695502 group and 63.1% in the bevacizumab RP group reported in this study. The median PFS for PF-06439535 versus bevacizumab-EU was 9.5 versus 7.7 months, similar to the 8.3 months and 9.0 months seen with BI 695502 and bevacizumab RP in this study. The median OS was 19.4 months for PF-06439535 versus 17.8 months for bevacizumab-EU; in the present study, the median OS was 15.6 months in the BI 695502 treatment group and 19.5 months in the bevacizumab RP group. Similar data were seen when ABP 215 (Mvasi) was compared with bevacizumab RP in a similar setting: the ORR was 39.0% versus 41.7%; the median PFS was approximately 7 months versus 8 months (estimated from Kaplan–Meier curves); the median OS was not reached in either group.^28^

The ORRs with BI 695502 and bevacizumab RP in the present study were similar to those observed in a previous study of bevacizumab plus carboplatin-paclitaxel in Japanese patients with advanced NSCLC.^9^ The ORR was 60.7% with carboplatin-paclitaxel plus bevacizumab and 31.0% with carboplatin-paclitaxel alone (*p* = 0.0013). However, lower ORRs of between 30% and 40% were reported with bevacizumab in three other NSCLC studies conducted across numerous different countries.^8^^,^^10^^,^^12^ The median PFS in the current study (8–9 mo) was slightly higher than previously observed with bevacizumab RP (6–7 mo).^9^^,^^10^^,^^12^ The median OS in the present study was 15 to 20 months, similar to the range of 12 to 18 months in three previous studies.^8^^,^^11^^,^^12^ However, a longer median OS of 23 months was reported by Niho et al.^9^ Minor variations between patient populations, approaches to clinical management, and assessment methods, which might not be obvious from the study publications, could explain the observed differences in efficacy end points. For example, studies have found that survival outcomes differ between individuals of Asian ethnicity/Japanese race versus non-Asian individuals.^29^, ^30^, ^31^, ^32^

In this study, 1.2% and 2.4% of patients tested positive for ADAs against BI 695502 and bevacizumab RP, respectively, and there were no positive tests for nAbs. Similar results have been reported in other studies of bevacizumab, with ADA rates of 1.4% to 2.5% reported in studies of healthy volunteers and patients with NSCLC.^27^^,^^28^^,^^33^

A range of AEs has previously been reported as class effects of drugs targeting the VEGF pathway.^34^, ^35^, ^36^, ^37^, ^38^, ^39^, ^40^ Of these, hypertension and proteinuria were the only two reported in at least 10% of patients in either arm of the present study, with similar incidences in both treatment groups. The proportion of patients with GI perforations and pulmonary hemorrhage, two other events previously identified as class effects of VEGF inhibitors, were low in our in both arms.

This study has several strengths. Patients with previously untreated advanced NSCLC represent a sensitive population for evaluation of potential bevacizumab biosimilar candidates owing to the large magnitude of benefit of bevacizumab RP in this patient population.^12^^,^^41^ Furthermore, the primary end point (ORR) is influenced by subsequent treatments and other confounding factors to a lesser extent than survival-based end points (e.g., OS), meaning that the principal study results are more robust. The choice of backbone chemotherapy represents another strength of the study, as it provides similarity with landmark studies of bevacizumab and allows a more meaningful comparison of the results with historical data.^8^^,^^9^^,^^12^ The baseline demographic and disease characteristics of the study population are consistent with patients with NSCLC encountered in the clinic, and the treatment regimen (for bevacizumab RP) is in line with typical clinical practice. Consequently, we consider the study results to be broadly applicable in real-world clinical settings. It is unfortunate that the duration of maintenance therapy with BI 695502 was curtailed in 13% of patients by the treatment switch. However, this did not affect the primary analysis and all the main study objectives were fulfilled. The lack of statistical comparison of certain end points (e.g., AE incidence rates) was a limitation of the study, meaning that the statistical significance of some between-group differences remains undetermined. Patient numbers were too small for definitive analysis of low-incidence AEs.

In conclusion, this study demonstrated that 18 weeks of treatment with either BI 695502 or bevacizumab RP provided equivalent ORR in patients with advanced nonsquamous NSCLC. Many of the secondary study end points, relating to efficacy, safety, immunogenicity, and PK, also exhibited similarities between the two drugs. These results enrich the evidence that bevacizumab biosimilars are a safe and efficacious alternative to bevacizumab RP.

## CRediT Authorship Contribution Statement

**Edward S. Kim:** Conceptualization, Formal analysis, Investigation, Writing - review & editing.

**Sigrid Balser:** Methodology, Formal analysis, Writing - review & editing.

**Klaus B. Rohr:** Conceptualization, Formal analysis, Methodology, Writing - review & editing.

**Ragna Lohmann:** Investigation, Project administration, Supervision, Writing - review & editing.

**Bernd Liedert:** Conceptualization, Formal analysis, Investigation, Methodology, Project administration, Writing - review & editing.

**Dorothee Schliephake:** Conceptualization, Investigation, Methodology, Writing - review & editing.

## Data Sharing Statement

To ensure independent interpretation of clinical study results, Boehringer Ingelheim grants all external authors access to relevant material, including participant-level clinical study data, as needed by them to fulfill their role and obligations as authors under the ICMJE criteria. Clinical study documents and participant clinical study data are available to be shared on request after publication of the primary manuscript in a peer-reviewed journal, and if regulatory activities are complete and other criteria met as per the BI Policy on Transparency and Publication of Clinical Study Data (see https://www.mystudywindow.com/us/).

Bona fide, qualified scientific and medical researchers are eligible to request access to the clinical study data with corresponding documentation describing the structure and content of the datasets. Upon approval, and governed by a Legal Agreement, data are shared in a secured data-access system for a limited period of 1 year, which may be extended upon request. Prior to providing access, clinical study documents and data will be examined, and, if necessary, redacted and de-identified, to protect the personal data of study participants and personnel, and to respect the boundaries of the informed consent of the study participants.

Researchers should use the https://vivli.org/ link to request access to study data and visit https://www.mystudywindow.com/us/ for further information.
