# Supplementary material for: Phase 3 Trial of BI 695502 Plus Chemotherapy Versus Bevacizumab Reference Product Plus Chemotherapy in Patients With Advanced Nonsquamous NSCLC
Source: JTO Clin Res Rep. 2021 Oct 28;3(1):100248. doi: 10.1016/j.jtocrr.2021.100248 (PMC8713120; doi:10.1016/j.jtocrr.2021.100248)
Supplement: Supplementary Materials [file mmc1.docx]

## Supplementary Materials

*Comprehensive Inclusion and Exclusion Criteria*

Patients were eligible for inclusion if they fulfilled each of the following criteria:

1. Males and females aged ≥18 years (for Japan only: aged ≥20 years at screening) with histologically or cytologically confirmed non-small-cell lung cancer (NSCLC). Mixed tumors were to be categorized according to the predominant histology. NSCLC was to be predominantly non-squamous.

2. Recurrent or metastatic disease (Stage IV) with an indication for therapy with paclitaxel, carboplatin and bevacizumab.

3. All patients were to sign and date an informed consent form consistent with International Conference on Harmonisation Good Clinical Practice guidelines and local legislation prior to participation in the trial (i.e. prior to any trial procedures, which included medication washout and restrictions) and were to be willing to follow the clinical trial protocol.

4. Patients harboring tumors without activating *epidermal growth factor receptor* (*EGFR*) mutation. Patients with unknown or activating *EGFR* mutation could have been included provided chemotherapy was the site standard of care. Despite positive *EGFR* mutational status, patients could enter the trial if the site’s best standard of care was to administer a chemotherapy regimen for that specific patient. However, if an *EGFR* test result was pending, and chemotherapy treatment would have been switched in case of a positive result, patients were not to be included in the trial.

5. Patients harboring tumors without activating *anaplastic lymphoma kinase* (*ALK*) mutation. Patients with unknown or activating *ALK* mutation could have been included provided chemotherapy was the site’s standard of care. Despite *ALK* mutational status, patients could have entered the trial if the site’s best standard of care was to administer a chemotherapy regimen for that specific patient. However, if an ALK test result was pending, and chemotherapy treatment would have been switched in case of a positive result, patients were not to be included in the trial.

6. At least one measurable lesion according to Response Evaluation Criteria in Solid Tumors, version 1.1 based on independent central review.

7. Eastern Cooperative Oncology Group performance status 0 or 1.

8. Adequate hepatic, renal, and bone marrow function:

a. Alanine aminotransferase (ALT) or aspartate aminotransferase (AST) ≤2.5 × upper limit of normal (ULN). If liver metastases were present, ALT or AST ≤5 × ULN.

b. Alkaline phosphatase ≤2.5 × ULN (≤5 × ULN in the presence of hepatic and/or bone metastases).

c. Serum bilirubin ≤1.5 × ULN, except in the case of known Gilbert’s syndrome.

d. Serum creatinine ≤1.5 × ULN or a creatinine clearance of ≥50 mL/min calculated by Cockcroft-Gault formula.

e. Proteinuria <2 g in 24 hours or an equivalent protein/creatinine ratio of <2000 mg/g creatinine (or <226 mg/mmol creatinine).

f. Absolute neutrophil count >1.5 × 10^9^/L.

g. Platelet count >100 × 10^9^/L.

h. Hemoglobin ≥9 g/dL (without transfusion within 2 weeks prior to randomization).

9. International normalized ratio ≤1.4 as analyzed locally. Partial thromboplastin time within normal limits according to local practice. Central laboratory analysis was used for coagulation parameters where local analysis was not available.

10. Life expectancy >6 months based on clinical investigator’s judgment.

11. For participants of reproductive potential (males and females), use of a medically acceptable method of contraception during the trial, i.e. a combination of two forms of effective contraception (defined as hormonal contraception, intrauterine device, condom with spermicide, etc). All patients (males and females of childbearing potential) were also to agree to use an acceptable method of contraception (see above) for 6 months following completion or discontinuation from the trial medication. A list of contraception methods meeting these criteria was provided in the patient information. Females were defined as of childbearing potential if they had not undergone a permanent contraceptive operation or they were not postmenopausal. Permanent contraceptive operation was defined as: hysterectomy, hysterosalpingectomy, or bilateral oophorectomy. The status of a female was considered as postmenopausal when she had not had a menstrual period for 12 consecutive months without an alternative medical cause.

Patients were excluded if they met any of the following criteria:

1. Prior therapy with mAbs or small-molecule inhibitors against vascular endothelial growth factor (VEGF) or VEGF receptors, including bevacizumab.

2. Prior systemic therapy for metastatic disease.

3. Prior systemic anticancer therapy or radiotherapy for locally advanced non-squamous NSCLC if completed <12 months prior to screening.

4. Patients who had results pending for *EGFR*/*ALK* mutation status, to the Investigator’s knowledge.

5. Previous malignancy other than NSCLC in the last 5 years except for basal cell cancer of the skin or pre-invasive cancer of the cervix.

6. Patients with known symptomatic brain metastasis:

a. Brain metastasis which was symptomatic at screening or randomization visits, or

b. Patients who had previously irradiated brain metastasis that had not been shown to be stable ≥1 month after completion of the radiation therapy (either by CT scan or MRI) at screening visit.

7. Diagnosis of small cell carcinoma of the lung, squamous cell carcinoma of the lung, NSCLC not specified or NSCLC not otherwise specified.

8. Patients with tumor/metastases cavitation, or invading into large blood vessels.

9. Patients with tumor/metastases close to large blood vessels that may have had an increased risk of bleeding, according to Investigator’s judgment.

10. Any unresolved toxicity Common Toxicity Criteria Grade >1 (except alopecia) from previous anticancer therapy (including radiotherapy).

11. History or evidence of inherited bleeding diathesis or coagulopathy with the risk of bleeding. Clinically non-significant minor bleeding was acceptable.

12. A thrombotic or hemorrhagic event ≤6 months prior to screening (includes hemoptysis, gastrointestinal (GI) bleeding, hematemesis, central nervous system hemorrhage, epistaxis, vaginal bleeding, cerebral infarction, transient ischemic attacks, myocardial infarction, angina, and coronary artery disease).

13. Current or recent (within 10 days of first dose of BI 695502/bevacizumab reference product [RP]) regular use of aspirin (>325 mg/day) or other non-steroidal anti-inflammatory drugs with antiplatelet activity or treatment with dipyridamole, ticlopidine, clopidogrel, or cilostazol.

14. Current treatment with oral, inhaled, or topical corticosteroids; the dose was not to exceed 10 mg/day prednisolone or equivalent. During the 4 weeks prior to Day 1, the dose was to be stable.

15. Intravenous, intramuscular, intra-articular, or parenteral corticosteroids within 6 weeks prior to Day 1 or throughout the trial, unless used for paclitaxel infusion premedication, according to regular institutional practice.

16. Current or recent (within 10 days of first dose of BI 695502/bevacizumab RP) use of full dose oral or parenteral anticoagulants or other thrombolytic agents for therapeutic (as opposed to prophylactic) purposes, clinically serious (as judged by the Investigator) non-healing wounds, or incompletely healed bone fracture.

17. Live/attenuated vaccine within 12 weeks prior to the screening visit.

18. History of myocardial infarction (≤6 months prior to screening), unstable angina, New York Heart Association Grade 2 or greater congestive heart failure, or serious cardiac arrhythmia requiring medication.

19. Patients with a history of poorly controlled hypertension or with resting blood pressure >150/100 mmHg in the presence or absence of a stable regimen of antihypertensive therapy.

20. Any surgical procedure within 28 days prior to the first dose of BI 695502/bevacizumab RP or anticipated elective surgery during the trial.

21. History of active gastroduodenal ulcer(s).

22. History of abdominal fistula as well as non-GI fistula, GI perforation or intra-abdominal abscess within 6 months prior to screening.

23. Active or chronic hepatitis B or C, ongoing HIV infection, or tuberculosis. Screening for HIV and tuberculosis (TB; PPD or QuantiFERON) was to be performed according to local practice and local regulatory guidance. There was to be no radiographic or clinical evidence of active TB.

24. Treatment within a clinical trial within 4 weeks prior to initiation of trial treatment. Patients who had received treatment with a drug that had not received regulatory approval for any indication within 4 weeks or a minimum of 5 half-lives, whichever was longer, of the initial dose of trial medication.

25. Patient considered unsuitable for inclusion by the Investigator (e.g. inability to understand and/or comply with study requirements or presence of any condition which, in the opinion of the Investigator, would not have allowed safe participation in the trial).

26. Pregnant or lactating women.

27. Known hypersensitivity to any of the trial drugs or their excipients.

**Supplementary Table 1.** Sensitivity analyses of the primary efficacy endpoint

| Assessment method | Population | Equivalence margin; CI for analysis | Result: estimated ratio of best ORR (CI) |
| --- | --- | --- | --- |
| PPS | PPS | 0.736–1.359; 90% | 0.879 (0.793–0.976) |
| 95% CI and comparing to different equivalence margin | FAS | 0.727–1.376; 95% CI compared to margins | 0.855 (0.754–0.970) |
| Multiple imputation for missing at random | FAS | 0.736–1.359; 90% | 0.874 (0.789–0.970) |
| Primary model + number of cycles | FAS | 0.736–1.359; 90% | 0.985 (0.902–1.076) |
| Primary model + region | FAS | 0.736–1.359; 90% | 0.852 (0.767–0.947) |

CI, confidence interval; FAS, full analysis set; ORR, overall response rate; PPS, per protocol set.

**Supplementary Figure 1.** Study design.


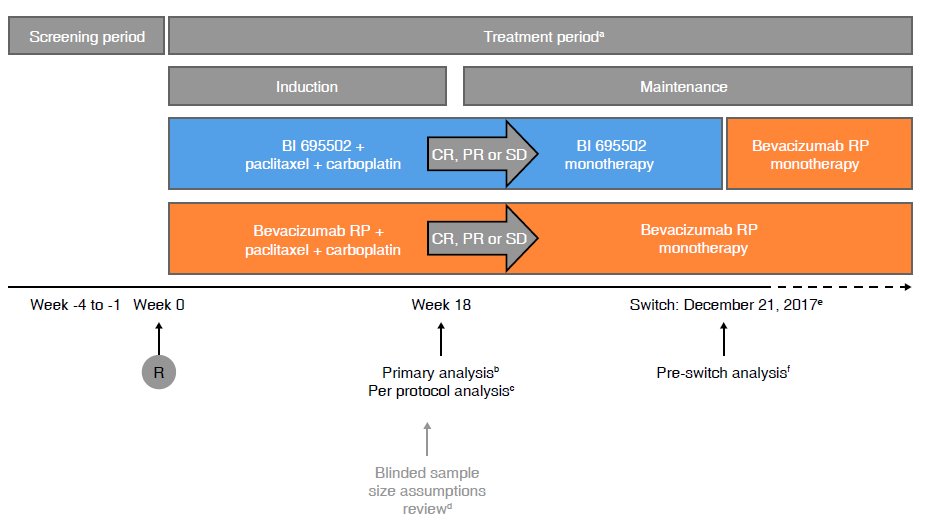


^a^Combination therapy induction cycles every 3 weeks for up to six cycles (18 weeks), followed by maintenance monotherapy until disease progression, unacceptable toxicity, withdrawal of consent or death.

^b^The primary efficacy analysis was performed when the central imaging review of Week 18 tumor response assessments for all patients had been completed, or earlier if no more patients were expected to complete the Week 18 Visit.

^c^Conducted as per the primary analysis, in individuals who did not experience any important protocol violations.

^d^Blinded sample size assumptions review started when approximately 100 patients per arm had had tumor response assessments performed until Week 18.

^e^In a deviation from the planned trial design, on December 21, 2017, following a manufacturing issue in a single batch of BI 695502, patients were switched from BI 695502 to bevacizumab RP. If no bevacizumab RP was immediately available, investigators could temporarily allow continuation on BI 695502.

^f^Secondary efficacy endpoints and, unless otherwise stated, safety data were derived from pre-switch data only.

CR, complete response; PR, partial response; R, randomization; RP, reference product; SD, stable disease.

**Supplementary Figure 2.** Pre-infusion plasma concentrations over time (pre-switch period).


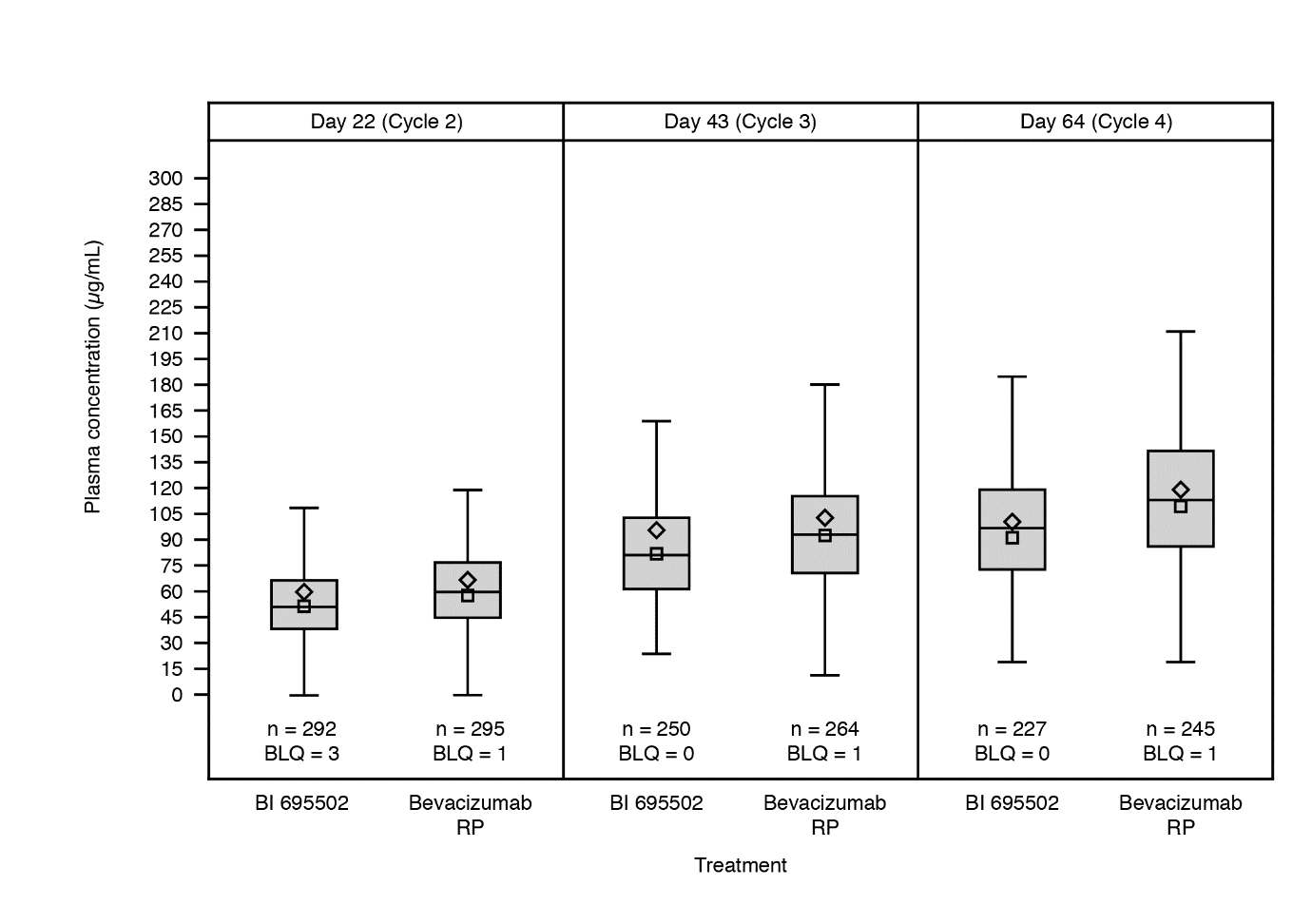
Data shown are the median (horizontal line) within the 25th percentile to 75th percentile box, geometric mean (square) and mean (diamond). The range shown by the ‘whiskers’ is defined by the 25th percentile minus 1.5 times the interquartile range (IQR) and the 75th percentile plus 1.5 times the IQR. Thus, outlying results are not shown.

BLQ, below limit of quantification; RP, reference product.
